# Visual performance, light distortion and patient reported outcomes with a new bi-aspheric non-diffractive extended depth of focus intraocular lens

**DOI:** 10.3389/fmed.2025.1513803

**Published:** 2025-02-26

**Authors:** Santiago Tañá-Sanz, Pedro Tañá-Sanz, Belén Elvira-Giner, Paz Orts-Vila, Pedro Tañá-Rivero

**Affiliations:** Department of Cataract Surgery, Oftalvist Alicante, Alicante, Spain

**Keywords:** extended depth of focus, non-diffractive, intraocular lens, phacoemulsification, cataract

## Abstract

**Background:**

To evaluate refractive, visual, and patient-reported outcomes three months after bilateral implantation of a novel bi-aspheric, non-diffractive extended depth of focus (EDOF) intraocular lens (IOL) using PhaseRing technology to achieve good vision across distances with reduced dysphotopsia.

**Methods:**

Twenty-two patients received bilateral Asqelio EDOF IOLs (AST VisionCare Inc.) and were evaluated 3 months post-surgery. The main outcomes assessed were refractive error, monocular and binocular visual acuities at distance, intermediate (67 cm) and near (40 cm), low contrast visual acuity, defocus curves, contrast sensitivity, and patient questionnaires.

**Results:**

The average postoperative spherical equivalent was −0.31 ± 0.30 D. Astigmatism of ≤ 1.00 D was present in all eyes (100%, *n* = 44), with 75% (*n* = 33) showing astigmatism of ≤ 0.50 D. Every patient attained a corrected distance visual acuity (CDVA) of 20/25 or better and a distance-corrected intermediate visual acuity (DCIVA) of 20/32 or better. Contrast sensitivity met or exceeded normal levels under both photopic and mesopic conditions, with and without glare, except at 12 cycles per degree under mesopic conditions with glare. Light distortion index was comparable to published reports on monofocal IOLs and other non-diffractive EDOF IOLs, and lower than diffractive multifocal IOLs. Post-surgery, 90.9% (*n* = 20) of patients reported being satisfied with their vision. No significant visual symptoms were reported.

**Conclusion:**

Asqelio™ EDOF IOL offers an efficient design, providing good clinical outcomes for distance and intermediate vision, while some patients reach functional levels of near vision. Its non-diffractive design minimizes dysphotopsia and reduces light distortion compared to other presbyopia-correcting IOLs.

## 1 Introduction

Cataracts stand as a leading cause of blindness globally, with cataract surgery ranking among the most commonly performed procedures worldwide. After the extraction of the cataract, the implantation of an intraocular lens (IOL) is performed to compensate for the lost power of the extracted lens and at the same time correct the patient’s ametropia, which is done in some cases without waiting for the cataract to develop, in what is known as refractive lensectomy. The use of monofocal IOLs allows for the correction of the patient’s ametropia and can provide excellent vision at a single distance (typically far), but falls show at providing good vision at multiple distances (i.e., far and near). The introduction of bifocal IOLs in the 1990s revolutionized patient care by offering near vision correction alongside ametropia correction. Trifocal IOLs, emerging in the European market in 2012, further enhanced patient outcomes by incorporating an intermediate vision focus, reducing reliance on corrective eyewear across a broader range of distances.

The approval of the first extended depth of focus (EDOF) lens by the FDA in 2016 (1) marked a milestone in vision correction. Subsequently, various EDOF models have entered the international market. Numerous studies have since investigated the visual and refractive efficacy of this type of lenses (2–4). A meta-analysis contrasting trifocal and EDOF IOLs indicates that while trifocal IOLs offer performance in near vision, they tend to induce more photic phenomena (5). Variations in multifocal IOLs stem from their optical principles (6), needing enhanced optical performance in modern IOLs to optimize reading capabilities and enhance patients’ quality of life post-cataract surgery (7, 8).

The present study aims to assess the clinical performance, light distortion and patient reported outcomes three months after bilateral implantation of Asqelio™ EDOF IOL following cataract surgery or refractive lensectomy. This is the first report on the clinical outcomes after implantation with this new non-diffractive EDOF IOL design.

## 2 Material and methods

This prospective, single-arm observational post-marketing study was approved by the Ethics Committee of Hospital Clínico San Carlos in Madrid, Spain, and was conducted in accordance with the principles outlined in the Declaration of Helsinki. Written informed consent was obtained from all patients before their participation, and the potential consequences of the study were thoroughly explained. Study registration was also carried out at www.clinicaltrials.gov (registration number: NCT06229756).

Inclusion criteria included patients of at least 50 years of age who were submitted to cataract surgery seeking spectacle-independence and bilaterally implanted with Asqelio*™* EDOF IOL (model ELIO130C), transparent intraocular media, other than the cataract preoperatively, and a potential visual acuity of 20/25 or better. Exclusion criteria included preoperatory corneal astigmatism exceeding 0.75 D, patients not providing informed consent, patients with concomitant ocular conditions, previous corneal surgery or trauma, extremely shallow anterior chamber, non-age-related cataracts, pregnancy, rubella, and those currently participating in other clinical investigations or expecting to undergo another ocular surgery during the study period.

### 2.1 Intraocular lens

The Asqelio™ EDOF IOL is manufactured by AST VisionCare, Inc. (previously AST Products, Inc.) (Billerica, MA, USA) via its proprietary Phase-Ring*™* technology. It is a one-piece foldable posterior chamber, UV absorbing optical implantable lens with non-diffractive design for the correction of presbyopia. The lens features a bi-aspheric geometry, spherical aberration of −0.27 microns, 360-degree sharp edge and Phase-Ring™-structured design on its posterior surface to extend the depth of focus for intermediate to near distances while maintaining distance vision. It has a total diameter of 13.0 mm with an optical zone of 6.0 mm and is manufactured in a power range from −10.00 to +40.00 D in 0.50 D increments. Crafted from a hydrophobic acrylic soft material, characterized by its glistening-free properties, the lens has a refractive index of 1.5 and an Abbe number of 50.

### 2.2 Surgical procedure

A limbal incision of 2.2 mm was performed, followed by standard phacoemulsification using the Centurion^®^ Vision System (Alcon Labs Inc., Fort Worth, TX, USA). After removing the cataract and polishing the posterior capsule, the capsular bag was filled with 1.0% sodium hyaluronate (Provisc™, Alcon, Fort Worth, TX, USA) to maintain the capsular space and facilitate IOL implantation. All patients were prescribed moxifloxacin 5 mg/mL (Vigamox™; Alcon), prednisolone 10 mg/mL (Pred-Forte™; Allergan, Inc., Irvine, CA, USA), and diclofenac-Lepori 1 mg/mL, administered in tapering doses over the first four weeks after surgery.

### 2.3 Preoperative and postoperative assessment

Before surgery, patients underwent comprehensive ophthalmologic examinations, including slit-lamp evaluation, measurements of logMAR uncorrected distance visual acuity (UDVA) and corrected distance visual acuity (CDVA), subjective and objective refraction assessments, intraocular pressure measurement, funduscopy, corneal topography, and biometry using the IOLMaster^®^ 700 (Carl Zeiss Meditec AG, Jena, Germany). The Barrett Universal II and Hoffer Q formulas were utilized for intraocular lens (IOL) calculations. The dominant eye was targeted for emmetropia, while slight myopia was aimed for in the non-dominant eye.

Three months post-implantation, patients underwent postoperative evaluations. Standard ophthalmologic assessments, including refraction and slit-lamp biomicroscopy, were conducted. Specifically, monocular and binocular logMAR UDVA, CDVA, uncorrected intermediate visual acuity (UIVA), and distance-corrected intermediate visual acuity (DCIVA) at 60 cm, as well as uncorrected near visual acuity (UNVA) and distance-corrected near visual acuity (DCNVA) at 40 cm, all measured under photopic conditions. DCNVA was also assessed monocularly and binocularly under mesopic conditions (3 cd/m^2^). Early treatment diabetic retinopathy study (ETDRS) charts were used for the measurements. Monocular and binocular defocus curves were generated with best distance correction using the ETDRS chart located at 4 m under photopic conditions, covering vergences from +2.00 to −5.00 D in 0.50 D increments (including 0.25 D steps between 0 and ± 0.50 D). All data were presented as mean ± standard deviation (SD) and ranges.

Binocular contrast sensitivity was tested with distance correction under photopic conditions (85 cd/m^2^), both with and without glare, for spatial frequencies of 3, 6, 12, and 18 cycles per degree (cpd), and under mesopic conditions (3 cd/m^2^) for spatial frequencies of 1.5, 3, 6, and 12 cpd, using the Clinical Trial Suite^®^ (M&S Technologies, Inc., IL, USA). Log absolute contrast threshold values were determined for each patient, spatial frequency, and luminance level combination. Mean values and standard deviations were calculated, and corresponding contrast sensitivity values (log CS) were derived from these thresholds to plot the contrast sensitivity function.

Light distortion was determined using the Light Distortion Analyzer (LDA) system, first under monocular conditions and then binocularly. In this assessment, patients responded to small peripheral light stimuli presented around a central light source, providing feedback to the system. Based on these responses, the LDA calculated several indices that quantify the size and regularity of distortion surrounding the central light source—specifically, the distortion index, the radius of the best-fit circle, and the irregularity of the best-fit circle (9).

Patient-reported outcomes were gathered through questionnaires. The Catquest-9SF (10), a widely recognized 9-item questionnaire, was used to determine limitations in daily activities due to poor vision, selected for its documented responsiveness in cataract surgery. This questionnaire consists of nine items with four response options, ranging from 1 (“no difficulty/very satisfied”) to 4 (“very great difficulty/very dissatisfied”), along with an additional “cannot decide” option treated as missing data. Items labeled A and C1 to C7 focus on difficulty levels, while item B addresses patient satisfaction.

Subjective visual symptoms were assessed using a questionnaire based on a validated quality-of-vision instrument (11). This questionnaire explores the frequency, intensity, and bothersomeness of ten common visual symptoms: glare, halos, starbursts, foggy vision, blurred vision, distortion, double vision, fluctuations in vision, difficulty focusing, and difficulty judging distances or depth. To aid in understanding, patients were shown simulated images depicting each symptom. They were then asked to rate each symptom on frequency (from 1 “Never” to 4 “Very often”), intensity (from 1 “None” to 4 “Severe”), and bothersomeness (from 1 “None” to 4 “A lot”).

Adverse events were recorded based on both solicited inquiries and spontaneous patient comments, as well as from observations made by the investigator.

### 2.4 Sample size and statistical analysis

The study’s estimated sample size was determined using the highest standard deviation observed in the defocus curve of typical monocular visual acuity (12). Specifically, a standard deviation of 0.24 logMAR at +2.00 diopters blur, a 95% confidence interval, and a maximum allowable margin of error of 0.10 logMAR were applied, resulting in a minimum required sample size of 22 patients.

Categorical variables were presented as frequencies and percentages, while continuous variables were summarized using means and standard deviations. Cumulative histograms of postoperative refractive error and refractive cylinder were constructed to evaluate refractive accuracy. Additionally, a cumulative histogram of postoperative visual performance was generated to assess the efficacy of refractive correction.

## 3 Results

A total of 22 consecutive patients were enrolled in the study, with a mean age of 67.95 ± 8.40 years (ranging from 55 to 83 years); half of them were female (*n* = 11, 50%). The preoperative demographic characteristics are summarized in Table 1. Standardized graphs depicting refractive and visual acuity outcomes at the three-month follow-up were constructed in line with established reporting guidelines (13).

**TABLE 1:** Demographic characteristics of participants shown as means, standard deviations (SD) and ranges.

	Values
Patients (*n*)	22
Sex (male/female)	11/11
Age (y)	67.95 ± 8.40 (55 to 83)
Sphere (D)	0.17 ± 5.15 (−11.50 to 7.00)
Refractive cylinder (D)	−0.63 ± 0.52 (−2.50 to 0.00)
Spherical equivalent (D)	−0.14 ± 4.15 (−11.75 to 7.00)
IOP (mmHg)	16.52 ± 3.17 (11 to 24)
CDVA (logMAR)	0.12 ± 0.20 (0.00 to 1.20)
K1 (D)	43.23 ± 1.35 (40.01 to 45.93)
K2 (D)	43.74 ± 1.38 (40.37 to 46.12)
Corneal astigmatism (D)	0.51 ± 0.22 (0.00 to 0.74)
Axial length (mm)	23.91 ± 1.58 (21.01 to 28.03)
ACD (mm)	3.16 ± 0.49 (1.99 to 4.20)
CCT (μm)	557.80 ± 37.47 (498 to 644)
LT (mm)	4.59 ± 0.34 (3.57 to 5.36)
WTW (mm)	12.02 ± 0.36 (11.20 to 12.60)
IOL spherical power (D)	20.68 ± 5.47 (9.00 to 33.00)

IOP, intraocular pressure; CDVA, corrected distance visual acuity; K, keratometry; ACD, anterior chamber depth; CCT, central corneal thickness; LT, lens thickness; WTW, white-to-white; IOL, intraocular lens power.

**FIGURE 1:**
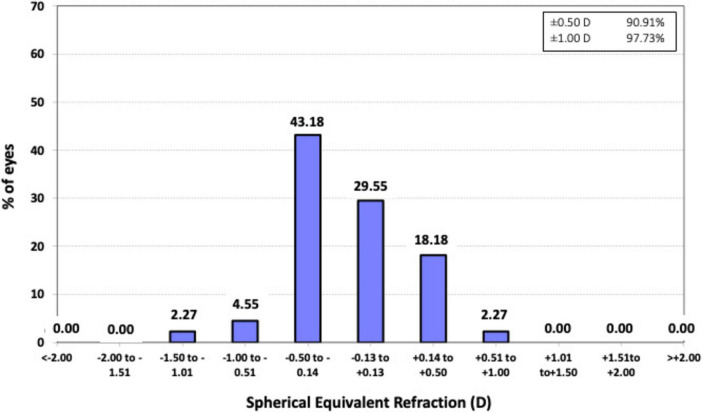
Histogram of postoperative spherical equivalent refractive accuracy 3 months after surgery relative to the intended target refraction.

**FIGURE 2:**
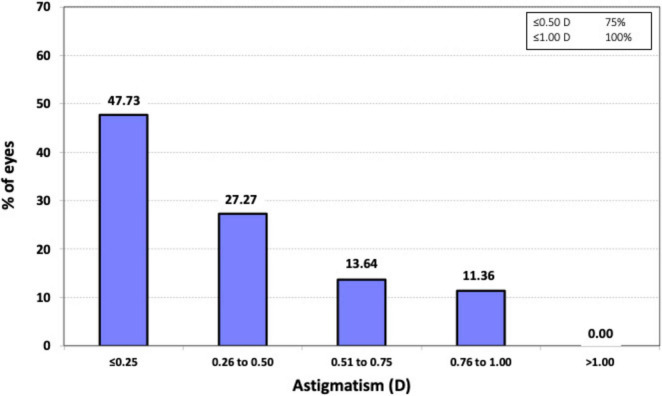
Histogram of the prevalence of postoperative refractive astigmatism at 3 months after surgery.

For the assessment of predictability, Figure 1 presents a histogram of postoperative spherical equivalent refraction relative to the intended target, while Figure 2 illustrates the postoperative refractive astigmatism. Concerning the spherical equivalent, the largest proportion of eyes, 43.18% (*n* = 19), fell within the range of −0.50 to −0.14 diopters (D), followed by 29.55% (*n* = 13) within the ± 0.13 D range, highlighting a high refractive accuracy, with the vast majority of patients achieving results close to the planned refraction. Overall, 97.73% (*n* = 43) of eyes were within ± 1.00 D of the target refraction, and 91% (*n* = 40) were within ± 0.50 D. The mean postoperative spherical equivalent was −0.31 ± 0.30 D, ranging from −1.50 D to +0.25 D.

Regarding astigmatism, all eyes (100%, *n* = 44) exhibited postoperative refractive cylinder values of ≤ 1.00 D, and 75% (*n* = 33) had values of ≤ 0.50 D. The mean postoperative refractive cylinder was −0.41 ± 0.33 D, with a range from 0 to −1.0 D.

**FIGURE 3:**
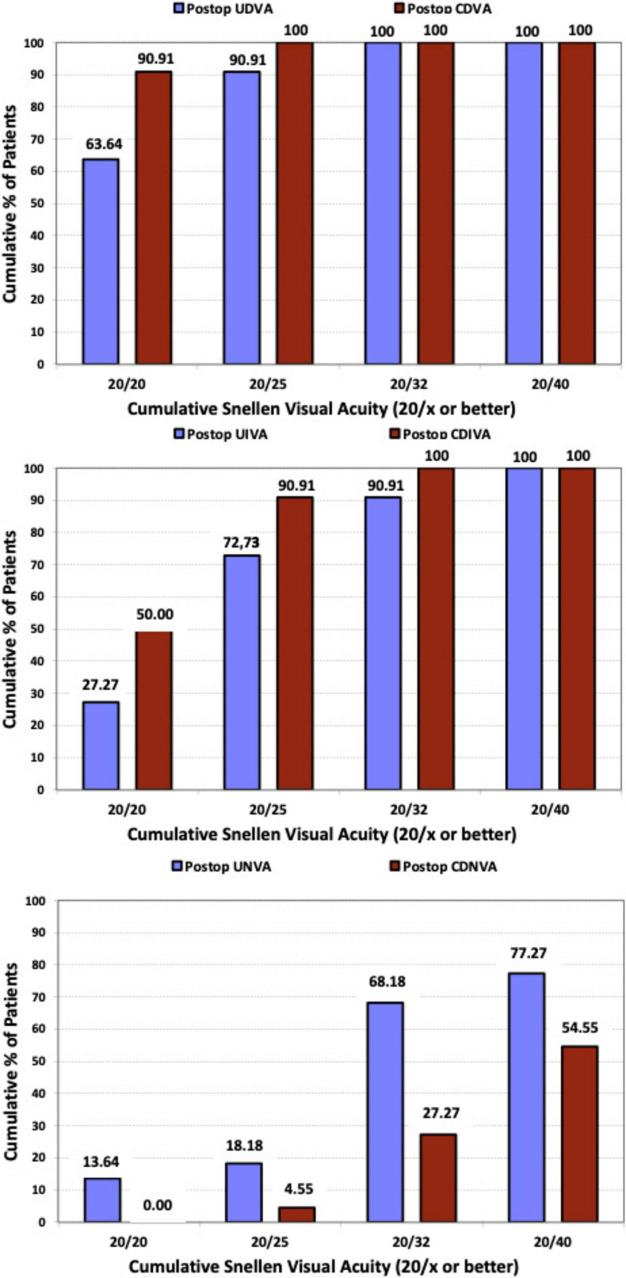
Cumulative proportion of patients having a given photopic binocular: uncorrected distance visual acuity (UDVA) and best-corrected distance visual acuity (CDVA) values **(upper)**; binocular uncorrected intermediate visual acuity (UIVA) and distance-corrected intermediate visual acuity (DCIVA) values **(middle)**; uncorrected near visual acuity (UNVA) and distance-corrected near visual acuity (DCNVA) values **(lower)**, at 3 months after surgery.

**FIGURE 4:**
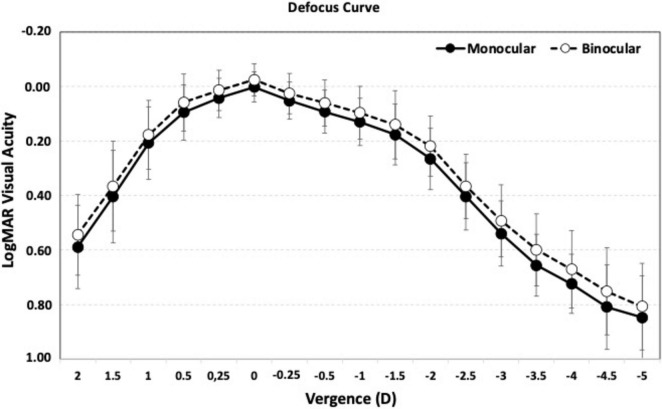
Mean, high-contrast, photopic, monocular and binocular logMAR visual acuity with best correction for distance, as a function of the chart vergence. Error bars represent standard deviation.

To evaluate the efficacy of the procedure, Figure 3 presents the cumulative postoperative binocular logMAR UDVA and CDVA (A), UIVA and DCIVA (B), and UNVA and DCNVA (C), respectively. All patients (100%) showed cumulative CDVA of 20/25 or better, and DCIVA of 20/32 or better. Specifically, 90.91% (*n* = 20) of patients showed an UDVA of 20/25 or better compared to 100% (*n* = 44) for CDVA, 72.73% (*n* = 16) of patients showed an UIVA of 20/25 or better compared to 90.91% (*n* = 20) for DCIVA, and 18.18% (*n* = 4) of patients showed an UNVA of 20/25 or better compared to 4.55% (*n* = 1) for DCNVA. Table 2 presents detailed measurements of visual acuity at different distances under both photopic and mesopic conditions. The monocular and binocular defocus curves with best correction for distance (Figure 4) show that visual acuity remained relatively stable across a wide range of defocus levels, indicating a smooth and extended depth of focus. The best performance was observed at 0.00 D (distance vision), with a gradual decline as defocus increased. The results show binocular vision maintained better performance than monocular vision at all vergences. A binocular visual acuity of 0.2 logMAR (20/32) or better was maintained up to −2.00 D of defocus, which corresponds to approximately 50 cm, demonstrating the lens’s ability to provide functional intermediate vision, and supporting the effectiveness of the lens in providing extended vision while maintaining good distance acuity.

**TABLE 2:** LogMAR visual acuity outcomes of patients implanted with the Asqelio EDOF intraocular lens shown as means, standard deviations (SD) and ranges.

	Monocular	Binocular
UDVA	0.10 ± 0.12 (−0.08 to 0.40)	0.02 ± 0.08 (−0.12 to 0.20)
CDVA	0.01 ± 0.06 (−0.08 to 0.18)	−0.03 ± 0.05 (−0.10 to 0.10)
UIVA	0.16 ± 0.13 (−0.10 to 0.40)	0.07 ± 0.10 (−0.10 to 0.30)
DCIVA	0.10 ± 0.11 (−0.10 to 0.40)	0.03 ± 0.08 (−0.10 to 0.20)
UNVA	0.30 ± 0.14 (0.00 to 0.54)	0.20 ± 0.13 (−0.04 to 0.40)
DCNVA	0.37 ± 0.12 (0.10 to 0.70)	0.31 ± 0.11 (0.10 to 0.50)
Low contrast CDVA	0.36 ± 0.12 (0.18 to 0.64)	0.29 ± 0.09 (0.20 to 0.50)
Low contrast DCIVA	0.41 ± 0.10 (0.20 to 0.70)	0.33 ± 0.11 (0.14 to 0.60)
Low contrast DCNVA	0.52 ± 0.15 (0.18 to 0.96)	0.45 ± 0.15 (0.12 to 0.70)

UDVA, uncorrected distance visual acuity; CDVA, corrected distance visual acuity; UIVA, uncorrected distance intermediate visual acuity; DCIVA, corrected distance intermediate visual acuity; UNVA, uncorrected distance near visual acuity; DCNVA, corrected distance near visual acuity.

**FIGURE 5:**
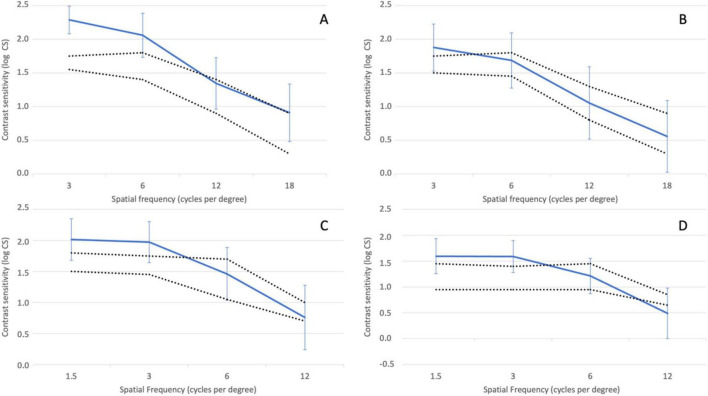
Contrast sensitivity function determined under photopic conditions (85 cd/m^2^) without **(A)** and with induced glare **(B)** and under mesopic conditions (3 cd/m^2^) without **(C)** and with induced glare **(D)** Dotted lines delimit the normal range for non-operated eyes above 60 years of age using the Functional Acuity Contrast Test (FACT) (11). Error bars represent the 95% confidence intervals.

Figure 5 illustrates the mean contrast sensitivity function measured under photopic conditions (85 cd/m^2^) with glare (A) and without glare (B), and under mesopic conditions (3 cd/m^2^) with glare (C) and without glare (D). Since the CTS system does not provide reference ranges for normal contrast sensitivity in healthy subjects under these conditions, for this analysis the normal ranges for non-operated eyes over 60 years old reported by Escaf et al. (14) using the Functional Acuity Contrast Test (FACT) were used. The results indicate that contrast sensitivity was within or above normal levels under both photopic and mesopic conditions, regardless of the presence of glare. The only exception was mesopic contrast sensitivity at 12 cycles per degree (cpd) with glare, where the mean value fell slightly below the normal range.

Regarding the questionnaires, the Catquest-9SF results (Table 3) showed that 90.9% of patients were either satisfied (13 out of 22) or very satisfied (7 out of 22) with their vision after surgery, with none reporting being very unsatisfied. This table presents the average scores and frequency of responses to questions related to difficulties in performing daily activities, as assessed by the Catquest-9SF. In most cases, the results indicate higher percentages for no difficulty (R4) in performing any of those activities [ranging from 63.6% (*n* = 14) to 90.9% (*n* = 20)], except for reading the newspapers, where half of the patients reported some difficulty and 40.91% (*n* = 9) reported no difficulty. Table 4 summarizes the results of the visual symptom questionnaire. No significant visual symptoms were reported in terms of frequency, intensity, or bothersomeness following the implantation of the Asqelio EDOF IOL. Halo was the only relevant visual symptom, with only 18.19% (*n* = 4) of patients reporting their presence quite often or very often, but none experiencing severe bothersome.

**TABLE 3:** Summary of patient-reported difficulties and satisfaction with their vision as per Catquest-9SF.

	Mean ± SD	Response frequencies (%)				
		R1	R2	R3	R4	R5
Do you find that your sight at present in some way causes you difficulty in your everyday life?	3.73 ± 0.55	0.00	4.55	18.18	77.27	0.00
Are you satisfied or unsatisfied with your current vision?	3.23 ± 0.61	0.00	9.09	59.09	31.82	0.00
**Do you have difficulty…**						
…Reading text in newspapers?	3.23 ± 0.87	9.09	0.0	50.00	40.91	0.00
…Recognizing the faces of people you meet?	3.73 ± 0.46	0.00	0.00	27.27	72.73	0.00
…Seeing the prices of goods when shopping?	3.68 ± 0.48	0.00	0.00	31.82	68.18	0.00
…Seeing to walk on uneven surfaces?	3.91 ± 0.29	0.00	0.00	9.09	90.91	0.00
…Seeing to do handicrafts, woodwork etc.?	3.68 ± 0.78	4.55	4.55	9.09	81.82	0.00
…Reading subtitles on TV?	3.64 ± 0.49	0.00	0.00	36.36	63.64	0.00
…Seeing to engage in an activity/hobby?	3.86 ± 0.35	0.00	0.00	13.64	86.36	0.00

Response coding: R1 (yes, extreme difficulty), R2 (yes, great difficulty), R3 (yes, some difficulty), R4 (no, no difficulty), R5 (cannot decide) for difficulties and R1 (very unsatisfied), R2 (fairly unsatisfied), R3 (fairly satisfied), R4 (very satisfied), R5 (cannot decide). SD, standard deviation.

**TABLE 4:** Summary of patient reported visual symptoms (mean score, type of symptom and frequency of responses) as per visual quality questionnaire.

		Frequency (%)			
	Mean ± SD	R1	R2	R3	R4
**Glare**					
Frequency	1.23 ± 0.43	77.27	22.73	0.00	0.00
Intensity	1.36 ± 0.79	77.27	13.64	4.55	4.55
Bothersome	1.18 ± 0.39	81.82	18.18	0.00	0.00
**Halo**					
Frequency	1.78 ± 0.89	54.55	27.27	13.64	4.55
Intensity	1.64 ± 0.79	54.55	27.27	18.18	0.00
Bothersome	1.32 ± 0.48	68.18	31.82	0.00	0.00
**Starburst**					
Frequency	1.32 ± 0.57	72.73	22.73	4.55	0.00
Intensity	1.36 ± 0.66	72.73	18.18	9.09	0.00
Bothersome	1.18 ± 0.39	81.82	18.18	0.00	0.00
**Hazy vision**					
Frequency	1.18 ± 0.39	81.82	18.18	0.00	0.00
Intensity	1.18 ± 0.39	81.82	18.18	0.00	0.00
Bothersome	1.23 ± 0.53	81.82	13.64	4.55	0.00
**Blurred vision**					
Frequency	1.14 ± 0.47	90.91	4.55	4.55	0.00
Intensity	1.14 ± 0.47	90.91	4.55	4.55	0.00
Bothersome	1.09 ± 0.29	90.91	9.09	0.00	0.00
**Distorted vision**					
Frequency	1.00 ± 0.00	100.00	0.00	0.00	0.00
Intensity	1.00 ± 0.00	100.00	0.00	0.00	0.00
Bothersome	1.00 ± 0.00	100.00	0.00	0.00	0.00
**Double vision**					
Frequency	1.00 ± 0.00	100.00	0.00	0.00	0.00
Intensity	1.00 ± 0.00	100.00	0.00	0.00	0.00
Bothersome	1.00 ± 0.00	100.00	0.00	0.00	0.00
**Fluctuation in vision**					
Frequency	1.09 ± 0.29	90.91	9.09	0.00	0.00
Intensity	1.09 ± 0.29	90.91	9.09	0.00	0.00
Bothersome	1.09 ± 0.29	90.91	9.09	0.00	0.00
**Difficulty focusing**					
Frequency	1.18 ± 0.38	81.82	18.18	0.00	0.00
Intensity	1.23 ± 0.53	81.82	13.64	4.55	0.00
Bothersome	1.14 ± 0.35	86.36	13.64	0.00	0.00
**DIFFICULTY perceiving distances/depth**					
Frequency	1.14 ± 0.64	95.45	0.00	0.00	4.55
Intensity	1.09 ± 0.43	95.45	0.00	4.55	0.00
Bothersome	1.05 ± 0.21	95.45	4.55	0.00	0.00

Response coding (frequency/severity/bothersome): R1 (never/none/none), R2 (occasionally/mild/a little), R3 (quite often/moderate/quite a bit), R4 (very often/severe/a lot). SD, standard deviation.

Light distortion parameters obtained under monocular and binocular conditions are displayed in Table 5.

**TABLE 5:** Light distortion parameters obtained under monocular and binocular conditions.

	Monocular (*n* = 33)		Binocular (*n* = 16)	
	Mean ± SD	Range	Mean ± SD	Range
LDI (%)	11.36 ± 5.47	5.33 to 25.47	8.51 ± 4.33	3.18 to 20.13
BFC radius	26.93 ± 6.23	18.7 to 41.3	23.24 ± 5.69	14.7 to 36.7
BFC irregularity	0.341 ± 0.24	0.01 to 1.07	0.40 ± 0.19	0.12 to 0.71

LDI, light distortion index; BFC, best fit circle; SD, standard deviation.

No adverse events were reported for any subject enrolled in the present study.

## 4 Discussion

EDOF IOLs are increasingly being implanted alongside trifocal lenses. They provide a continuous extended range of vision without generating specific focal points for particular distances, offering good VA results for distance and intermediate ranges comparable to multifocal IOLs, though with somewhat poorer near VA (15). The technologies used to create the extended range in these IOLs vary considerably among different platforms (3, 16). Due to this variability, the American Academy of Ophthalmology reached a consensus on the criteria for defining and evaluating the performance of EDOF IOLs (17). Based on these recommendations, the US Food and Drug Administration established several clinical criteria for EDOF IOLs in the American National Standards Institute (ANSI)/AAO Standard Z80.35–2018 (18).

According to these criteria, EDOF IOLs should provide a monocular depth of focus at 0.2 logMAR that is at least 0.50 diopters (D) greater than that of a monofocal control. Additionally, the monocular photopic DCIVA at 66 cm should exceed that of a monofocal control lens, with at least 50% of eyes achieving a VA of 0.2 logMAR or better. Finally, the mean monocular photopic CDVA should be non-inferior to that of a monofocal control IOL. Assessments of mesopic contrast sensitivity and visual symptom questionnaires are also required, although there are no specific criteria regarding visual quality or disturbances.

At the present moment, three IOLs in the EDOF IOL category may be considered as non-diffractive EDOF IOLs that do not use spherical aberration as source of EDOF but refractive elements to reshape the wavefront: Acrysof^®^ IQ Vivity^®^ (Alcon Inc., USA) (now also a version with the new Clareon material is available), Lucidis^®^ (SAV-IOL SA, Switzerland), and Asqelio™ EDOF (AST VisionCare, Inc., USA). The present study constitutes the first report on the clinical outcomes with the Asqelio™ EDOF IOL.

Analyzing the visual performance of Asqelio™ EDOF obtained in the present study, all patients (100%) showed cumulative CDVA of 0.1 LogMAR or better, and DCIVA of 0.2 LogMAR or better. Specifically, 90.91% of patients showed an UDVA of 0.1 LogMAR or better compared to 100% for CDVA, 72.73% of patients showed an UIVA of 0.1 LogMAR or better compared to 90.91% for DCIVA, and 18.18% of patients showed an UNVA of 0.1 LogMAR or better compared to 4.55% for DCNVA. The difference observed between uncorrected and corrected visual performance, particularly for near vergences, results from attempting a slight residual myopia in the non-dominant eye, known as mini-monovision, which has been reported to give good clinical outcomes with similar types of IOLs (19) and is common practice in the study center, aiming to expand the range of binocular clear vision. Considering this, the visual performance with the IOL is good under both monocular and binocular conditions.

In a sample of 20 patients implanted with the Acrysof^®^ IQ Vivity^®^ IOL, Sabur and Unsal (20) reported a mean monocular CDVA of 0.02 ± 0.04 LogMAR, DCIVA of 0.18 ± 0.09 and DCNVA of 0.30 ± 0.11LogMAR. These outcomes are comparable to those found in the present study, with mean values of 0.01 ± 0.06, 0.10 ± 0.11, and 0.37 ± 0.12 for monocular CDVA, DCIVA and DCNVA, respectively. With regards to binocular VA values, Sabur and Unsal (20) reported mean values of 0.01 ± 0.03, 0.13 ± 0.07 and 0.24 ± 0.10. Binocular VA values found in the present study were slightly better, −0.03 ± 0.05, 0.03 ± 0.08 and 0.20 ± 0.13 LogMAR for CDVA, DCIVA and DCNVA, respectively. In their study, they compared the outcomes of the non-diffractive EDOF IOL against the TECNIS Eyhance^®^ (Johnson & Johnson Surgical Vision, Inc, USA), categorized as an enhanced monofocal IOL. They concluded that visual performance for distance and intermediate vision was similar between both IOLs, with near vision being significantly better for the EDOF IOL.

van Amelsfort et al. (21) found that adjusting for emmetropia in the dominant eye and inducing slight myopia in the nondominant eye (-0.25 D to -0.50 D) led to improved near visual acuity, greater patient satisfaction, and higher levels of spectacle independence. Similarly, Newsom and Potvin (22) demonstrated that targeting -0.75 D of myopia in the nondominant eye resulted in an improvement of more than one line in near visual acuity compared to other studies focusing on bilateral emmetropia.

In a randomized double-blind comparison study between Acrysof^®^ IQ Vivity^®^ and TECNIS Symfony^®^ (Johnson & Johnson Surgical Vision, Inc, USA), a diffractive EDOF IOL, Scheepers and Hall (23) found binocular VA values of −0.03 ± 0.05, 0.04 ± 0.08 and 0.22 ± 0.12 LogMAR for CDVA, DCIVA and DCNVA, respectively, in patients implanted with the Vivity refractive EDOF 3 months after surgery, very much in agreement with those found in the present study with Asqelio™ EDOF IOL. These were not significantly different from those obtained in patients implanted with the Symfony IOL, which were, respectively, of −0.03 ± 0.05, 0.03 ± 0.10 and 0.26 ± 0.11 LogMAR.

Sabur and Unsal (20) reported binocular defocus curve outcomes with bilateral implantation of the non-diffractive Acrysof Vivity EDOF IOL. According to their mean binocular defocus curve, CDVA peaked at 0 D, with a progressive decline that reached 0.1 LogMAR around −1.25 D vergence and 0.2 LogMAR around −2.0 D. Similar profile was reported by Pantanelli et al. (24) comparing Acryso IQ Vivity to a monofocal control. They also showed two more lines of vision at intermediate and near vergences compared to the control IOL. In the present study, Asqelio™ EDOF IOL showed a similar behavior, with a mean binocular defocus curve that peaked at 0 D and had a similar progressive decline, reaching 0.2 LogMAR at −2.0 D.

According to the literature, eyes implanted with an EDOF IOL should experience fewer dysphotopsia and less loss of contrast sensitivity compared with those fitted with a conventional multifocal IOL (25–28). Sabur and Unsal (20) obtained similar contrast sensitivity outcomes in Acrysof^®^ IQ Vivity^®^ patients and Eyhance^®^ enhanced monofocal IOL, with differences not being statistically significant at any spatial frequency or light condition. In the present study, contrast sensitivity values obtained with Asqelio™ EDOF showed either within or above normal range under both photopic and mesopic conditions, both with and without glare being induced. Given that the CTS system does not provide with a reference range of normality for healthy subjects under photopic and mesopic conditions with and without glare, it must be noted that the normal ranges for non-operated eyes above 60 years of age used by Escaf et al. (14). using the Functional Acuity Contrast Test (FACT) were used as a reference here.

With regards to light distortion, Guarro et al. (29) concluded that diffractive EDOF IOL models induced similar visual disturbances that were worse than those produced by the non-diffractive EDOF model. In their study they found glare, halos, and starbursts to be similar between the non-diffractive EDOF IOL studied, AcrySof^®^ IQ Vivity^®^, and a monofocal control IOL. The mean LDI values they obtained for the non-diffractive EDOF IOL were 14.36 ± 10.25 (range 4.46 to 42.62) monocularly, and 8.24 ± 3.86 (3.82, 16.31) binocularly. These results are in agreement with those found in the present study in patients implanted with Asqelio™ EDOF, showing that the light distortion of Asqelio™ EDOF and Acrysof^®^ Vivity^®^ is very similar both monocularly and binocularly. These outcomes are considerably better than those reported in the literature using the same method with other presbyopia-correcting IOLs (30, 31).

Patient-reported outcomes show 90.9% of patients as satisfied or very satisfied with their vision after bilateral implantation of Asqelio™ EDOF, while none of the patients’ reports being very unsatisfied. The Catquest-9SF outcomes show no difficulty for performing most of the daily activities, except for reading the newspapers, where half of the patients reported some difficulty, as expected given the close distance range needed. Regarding visual disturbance symptoms, literature shows that diffractive EDOF IOLs perform worse than non-diffractive EDOF IOLs (19), and no significant differences in visual disturbances were found compared to a monofocal lens (29). In the study by Guarro et al. (29), the most frequent visual symptom reported by patients implanted with Acrysof^®^ IQ Vivity^®^ was starbursts, with 4.5% of patients reporting it as very frequent and 13.6% as moderately severe and quite bothersome. These were slightly different in the study by Pantanelli et al. (24), who found glare as the most frequent visual symptom, with 4.2% of patients reporting very frequent and very bothersome. In the study by Newsom and Potvin (22), halos and starbursts had increased frequency (18 and 39%, respectively), severity (33 and 55%, respectively), and bothersome (21 and 18%, respectively). In the present study, patient reported outcomes with Asqelio™ EDOF show halo as the most frequent visual symptom, with 4.5% of patients reporting as very frequent, but none of them considered it as bothersome.

## 5 Conclusion

To conclude, the present study supports that the Asqelio™ EDOF IOL is an efficient IOL design providing good clinical outcomes at distance and intermediate distances, while some patients may also reach functional near vision. Its non-diffractive design also benefits the lack of relevant dysphotopsia and reduced light distortion compared to other presbyopia-correcting IOL designs. The high level of patient satisfaction reported after implantation makes it a valuable option for patients seeking spectacle independence with no visual disturbance, and very good visual outcomes.

## Data Availability

The raw data supporting the conclusions of this article will be made available by the authors, without undue reservation.
